# Phase I dose-escalation study of [¹⁷⁷Lu]Lu-LNC1011, a long-circulating dansyl-modified PSMA theranostics, in metastatic castration-resistant prostate cancer

**DOI:** 10.7150/thno.128143

**Published:** 2026-03-04

**Authors:** Jiarou Wang, Rongxi Wang, Jialin Xiang, Hongzhang Yang, Tianzhi Zhao, Feng Guo, Yingkui Liang, Zhaohui Zhu, Jingjing Zhang

**Affiliations:** 1 Department of Nuclear Medicine, Peking Union Medical College Hospital, Chinese Academy of Medical Science and Peking Union Medical College; State Key Laboratory of Complex Severe and Rare Diseases, Beijing; Beijing Key Laboratory of Molecular Targeted Diagnosis and Therapy in Nuclear Medicine, Beijing, 100730, China.; 2 Department of Diagnostic Radiology, Yong Loo Lin School of Medicine, National University of Singapore, Singapore, 119074, Singapore.; 3 Theranostics Center of Excellence, Yong Loo Lin School of Medicine, National University of Singapore, Singapore, 138667, Singapore.; 4 Department of Nuclear Medicine, The Sixth Medical Center of PLA General Hospital, Beijing, 100048, China.; 5 Nanomedicine Translational Research Program, Yong Loo Lin School of Medicine, National University of Singapore, Singapore, 117597, Singapore.; 6 Clinical Imaging Research Centre, Centre for Translational Medicine, Yong Loo Lin School of Medicine, National University of Singapore, Singapore, 117599, Singapore.

**Keywords:** mCRPC, PSMA, [^177^Lu]Lu-LNC1011, Dansyl-Modified PSMA, Dosimetry, Phase I, Radiopharmaceutical therapy

## Abstract

**Rationale:**

[^177^Lu]Lu-D-Dan-Phe-PSMA ([^177^Lu]Lu-LNC1011) is a novel long-circulating prostate-specific membrane antigen (PSMA) therapeutic radioligand. This Phase I study aimed to investigate its safety, dosimetry, and initial treatment efficacy in metastatic castration-resistant prostate cancer (mCRPC) patients.

**Methods:**

This open-label, non-randomized, Phase I dose-escalation trial enrolled mCRPC patients with PSMA-positive lesions confirmed by PSMA PET/CT. A total of nine patients received [^177^Lu]Lu-LNC1011 at administered activity levels of 1.85 GBq, 2.78 GBq, and 3.70 GBq.

**Results:**

During the dose-limiting toxicity (DLT) observation period, no DLTs occurred in any activity group. The cumulative absorbed dose to organs increased with the injected activity. The effective dose to the whole body was 1.30E-01 ± 1.91E-02 mSv/MBq. The kidneys received the highest radiation dose (3.33 ± 1.09 Gy/GBq), consistent with the expected biodistribution of PSMA-targeted ligands. For salivary glands and red bone marrow, the absorbed doses were 1.70 ± 1.04 Gy/GBq and 0.11 ± 0.03 Gy/GBq, respectively. The mean absorbed dose to tumors was 12.29 ± 6.50 Gy/GBq. The effective half-life of [^177^Lu]Lu-LNC1011 was 49.44 ± 12.58 h in the whole body and 128.3 ± 62.79 h in tumor lesions. After two treatment cycles, the 3.70 GBq cohort (n = 3) demonstrated 100% PSA reduction (3/3), with 66.7% (2/3) achieving ≥50% PSA decline; simultaneously, Response Evaluation Criteria in PSMA PET/CT (RECIP) 1.0 assessment showed partial response (PR) in 66.7% (2/3) of patients and stable disease (SD) in 33.3% (1/3).

**Conclusion:**

[^177^Lu]Lu-LNC1011 was well tolerated across all dose levels, delivering high tumor-absorbed doses and prolonged tumor retention. The 3.70 GBq cohort demonstrated notable therapeutic efficacy. These favorable safety, dosimetry, and efficacy findings support the investigation of extended multi-cycle treatment with [¹⁷⁷Lu]Lu-LNC1011 at 3.70 GBq per cycle in future clinical studies.

## Introduction

Prostate cancer (PCa) is the most common cancer diagnosed in men and ranks as the second leading cause of cancer-related mortality in men 1, 2. Prostate-specific membrane antigen (PSMA) is a highly expressed antigen in prostate adenocarcinoma cells and has emerged as a promising target for theranostics in PCa 3, 4. PSMA-targeted Radiopharmaceutical Therapy (RPT), such as lutetium-177 (^177^Lu)-labeled PSMA ligands, has demonstrated significant survival benefits in patients with mCRPC in Phase II and III clinical trials 5-7. Consequently, [^177^Lu]Lu-PSMA-617 (Pluvicto™, lutetium Lu-177 vipivotide tetraxetan) was approved by the US Food and Drug Administration in 2022 for the treatment of mCRPC 8, 9. Its pharmacokinetic profile is characterized by rapid renal clearance of unbound radioligand, a feature that reduces off-target radiation exposure but may concurrently limit sustained intratumoral radiation delivery. This underscores the importance of developing next-generation agents that optimize the balance between tumor retention and systemic clearance to advance the therapeutic potential of RPT.

A promising strategy to improve the therapeutic efficacy of PSMA-targeted RPT might be to enhance tumor-target binding affinity and prolong intratumoral retention time. One strategy for incorporating an albumin-binding moiety into the original structure has shown considerable interest in prolonging circulation time and increasing tumor retention 10-15. Building on this concept, our team has developed two derivatives by conjugating truncated EB molecules to a DOTA chelator via a PSMA linker and labeling them with ^177^Lu to produce [^177^Lu]Lu-EB-PSMA and [^177^Lu]Lu-LNC1003 16-21. Early clinical evaluation of these ligands has shown substantially improved tumor retention compared to [^177^Lu]Lu-PSMA-617, albeit with the drawback of greater red marrow exposure. These findings underscore the double-edged nature of both the improved tumor retention and the safety considerations associated with albumin-binding modified PSMA ligands. Achieving a better balance is thus necessary to further optimize these compounds and balance tumor retention with bone marrow toxicity, thereby advancing PSMA theranostics.

[^177^Lu]Lu-D-Dan-Phe-PSMA ([^177^Lu]Lu-LNC1011) is a novel, long-circulating PSMA therapeutic probe incorporating a dansyl-based albumin-binding motif. Preclinical studies of [^177^Lu]Lu-LNC1011 exhibited a high binding affinity and notable tumor-to-nontarget contrast. The tumor uptake and Area Under Curve (AUC) values of [^177^Lu]Lu-LNC1011 were 7-8 times higher compared to [^177^Lu]Lu-PSMA-617 22. In the present study, we conducted a dose-escalation Phase I trial of [^177^Lu]Lu-LNC1011 in patients with mCRPC. The primary objectives of this study are to evaluate the safety of escalating activity groups of [^177^Lu]Lu-LNC1011, to investigate its biodistribution, organ dosimetry, and preliminary therapeutic efficacy in patients with mCRPC, providing a basis for activity selection in future large-scale single-dose and multi-cycle studies.

## Materials and Methods

### Study design

This study was approved by the Institutional Review Board of Peking Union Medical College Hospital (No. I-23PJ1515), and each patient signed the informed consent form. The study was also registered on ClinicalTrials.gov (NCT06250244).

The initial activity of [^177^Lu]Lu-LNC1011 was set at 1.85 GBq (Activity Group 1), based on previous preclinical studies and other clinical trials using albumin-binding PSMA agents. Subsequent cohorts received a dose escalation of 0.925 GBq from the previous cohort (2.78 GBq for Activity Group 2 and 3.70 GBq for Activity Group 3). A standard 3 + 3 dose-escalation design was employed with dose-limiting toxicity (DLT) defined as any ≥ Grade 3 hematologic toxicity lasting > 7 days, ≥ Grade 3 non-hematologic toxicity, or any Grade 4 toxicity occurring within the 42-day safety observation period. Adverse events (AEs) were graded using the Common Terminology Criteria for Adverse Events Version 5 (CTCAE 5.0).

### Eligibility

Aside from not requiring prior docetaxel treatment, the inclusion criteria—including molecular imaging selection requiring at least one lesion with PSMA PET SUV_max_ ≥ 20 and exclusion of FDG PET-positive/PSMA-negative lesions—were identical to those used in the *TheraP* trial 6. PET/CT scan protocols and detailed image analysis procedures are available in the supplemental materials.

### Radiopharmaceuticals

[¹⁷⁷Lu]Lu-LNC1011 was synthesized by reacting 50 μg LNC1011 with [¹⁷⁷Lu]LuCl₃ in ammonium acetate buffer (pH 5.5) at 95 °C for 30 min. The full synthesis protocol is detailed in Supplementary Materials.

### Imaging and dosimetry

For [¹⁷⁷Lu]Lu-LNC1011 dosimetry, planar whole-body scans and SPECT/CT were performed. Absorbed doses to organs were calculated using Hybrid-Dosimetry and OLINDA/EXM 2.2.0. Detailed imaging protocols and dosimetric procedures are provided in Supplementary Materials.

### Treatment regimen and follow-up

The treatment regimen was planned for up to 2 cycles, with an inter-cycle time interval of 6 weeks. A follow-up [⁶⁸Ga]Ga-PSMA-11 PET/CT scan was conducted six weeks after administration. The last safety laboratory assessments for DLT evaluation were performed at Day 42 (± 3) of each cycle. Detailed treatment regimens and follow-up procedures are provided in the Supplementary Materials.

### Outcomes

The primary endpoint of this study was to evaluate the safety of [^177^Lu]Lu-LNC1011. The secondary objectives included dosimetry and preliminary assessment of treatment efficacy after two cycles. PSA responses were evaluated according to the Prostate Cancer Clinical Trials Working Group-3 guidelines 23. Imaging assessments are conducted in accordance with the RECIST 1.1 24 and RECIP 1.0 25. The disease control rate (DCR) is defined as the proportion of patients with complete response (CR), partial response (PR), or stable disease (SD). The objective response rate (ORR) includes only the proportion of patients with CR and PR.

### Statistical analysis

Statistical analyses were conducted using SPSS software (Version 26.0, IBM Corp., NY, USA) and GraphPad Prism (Version 10.0.3, GraphPad Software, Armonk, Boston, MA, USA). The quantitative data are presented as mean ± standard deviation. A significance level of P < 0.05 was deemed statistically significant.

## Results

### Patient characteristics

A total of 9 patients were enrolled between May 2023 and December 2024. The mean age of these patients was 72 ± 5 y, and the mean PSA level at baseline was 149.61 ± 303.28 ng/mL. The patients' detailed characteristics were summarized in **Table 1.**

### Safety and side effects

Following the administration of two cycles of [^177^Lu]Lu-LNC1011, there was no immediate or life-threatening serious adverse effect observed. During the 42-day follow-up period, no instances of hepatic or renal toxicity were observed in any of the activity groups. Additionally, one patient who had pre-existing hepatic abnormalities at baseline did not show any significant changes in indicators during treatment.

Throughout the treatment period, hematological toxicity was observed to be manageable. In the 1.85 GBq group, there was one case (patient 2) of Grade 1 anemia after two treatment cycles (33.33%, 1/3), and two cases (patient No. 1 and No. 2) of Grade 1 leukopenia (66.67%, 2/3) after one treatment cycle, with no significant changes in the second treatment cycle.

In the 2.78 GBq group, 3 cases (patients No. 4, 5, and 6) of Grade 1 anemia were observed at baseline (100.00%, 3/3), with one patient (patient No. 4) returning to normal levels during treatment. Additionally, one case (patient No. 4) of Grade 1 leukopenia (33.33%, 1/3) occurred after one treatment cycle, with no significant changes in the second treatment cycle.

In the 3.70 GBq group, 2 cases (patient No. 8 and No. 9) of Grade 2 anemia were present at baseline (66.67%, 2/3), with no further decrease during treatment. Notably, one patient (patient No.7) developed Grade 1 thrombocytopenia after one treatment cycle (33.33%, 1/3), and an additional 42-day follow-up revealed progression to Grade 3 thrombocytopenia. This patient had a baseline platelet count of 143 × 10⁹/L. After one cycle, the platelet count decreased to 86 × 10⁹/L and further to 32 × 10⁹/L by week 8. However, the patient exhibited no significant signs of hemorrhage. Additionally, at the 12-week follow-up, the patient's platelet count was 40 × 10⁹/L and subsequently stabilized without further decline. The adverse effect details for all activity groups are summarized in **Table 2**.

### Biodistribution and dosimetry

**Figure 1:**
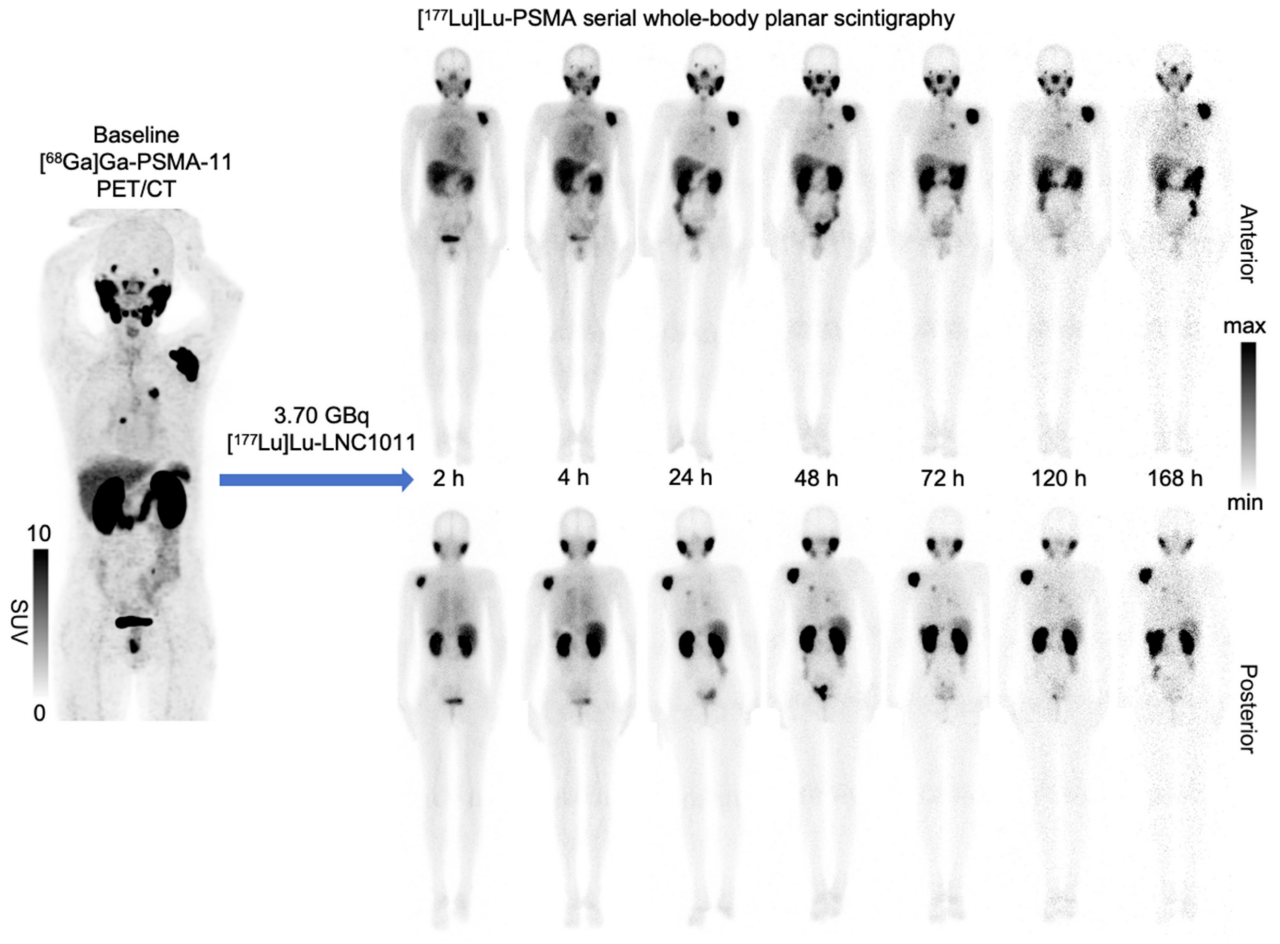
Whole-body scintigraphy (anterior and posterior views) obtained at 2, 24, 48, 72, and 168 h after administration of 3.70 GBq of [¹⁷⁷Lu]Lu-LNC1011 in a 65-year-old patient (Patient 7).

**Figure 2:**
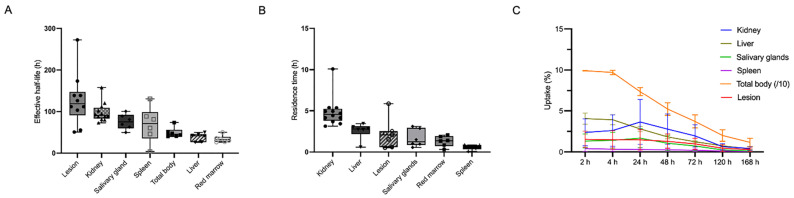
(A-B) Effective half-life (h) and residence time (h) of [177Lu]Lu-LNC1011. (C) Organ, total-body and lesion uptakes as percentages of the administered activity (%AA) over time.

The biodistribution study and dosimetry analysis were based on the post-injection images in three activity groups. The biodistribution of [^177^Lu]Lu-LNC1011 is illustrated in **Figure 1**, with notable physiological uptake in the lacrimal glands, salivary glands, liver, spleen, and kidneys. Biodistribution analysis confirmed primary renal clearance, with kidney uptake declining from 4.03 ± 2.97 %ID at 24 h p.i. to 0.47 ± 0.18 %ID by 168 h (**Figure 2**). Salivary glands demonstrated moderate early uptake (2 h: 1.26 ± 0.79 %ID) but washed out substantially, declining to 0.12 ± 0.07 %ID at 168 h. Uptake in the liver and spleen remained low throughout, consistent with a minor hepatobiliary contribution. One key observation was differential washout kinetics: non-target organs, such as salivary glands, liver, and spleen, showed rapid decline to near-background levels by the final imaging time point, whereas tumor lesions retained substantially higher uptake. This contrast indicates the tracer's favorable tumor retention and efficient clearance profile in the normal tissues. The effective half-life, derived from time-activity curves (TACs) of [^177^Lu]Lu-LNC1011, was 49.44 ± 12.58 h in the total body and 128.3 ± 62.79 h in tumor lesions. The effective half-lives for other organs are provided in the Supplementary Materials. The residence times were 6.18 ± 1.94 h for kidneys, 3.25 ± 0.70 h for liver, 1.63 ± 0.94 h for salivary glands, and 1.27 ± 0.23 h for red bone marrow. Effective half-lives, residence time, and TAC, including normal organs and the tumor, were illustrated in **Figure 2A-C**. For salivary glands and red bone marrow, the average absorbed dose was 1.70 ± 1.04 Gy/GBq and 0.11 ± 0.03 Gy/GBq, respectively. The kidneys received the highest absorbed dose, estimated at 3.33 ± 1.09 Gy/GBq. The effective dose for the whole body was 0.10 ± 0.02 mSv/MBq, and the average absorbed dose in tumors was 12.29 ± 6.50 Gy/GBq. The absorbed doses of other organs in the three dosage groups are summarized in **Table 3**.

### Treatment efficacy

**Figure 3:**
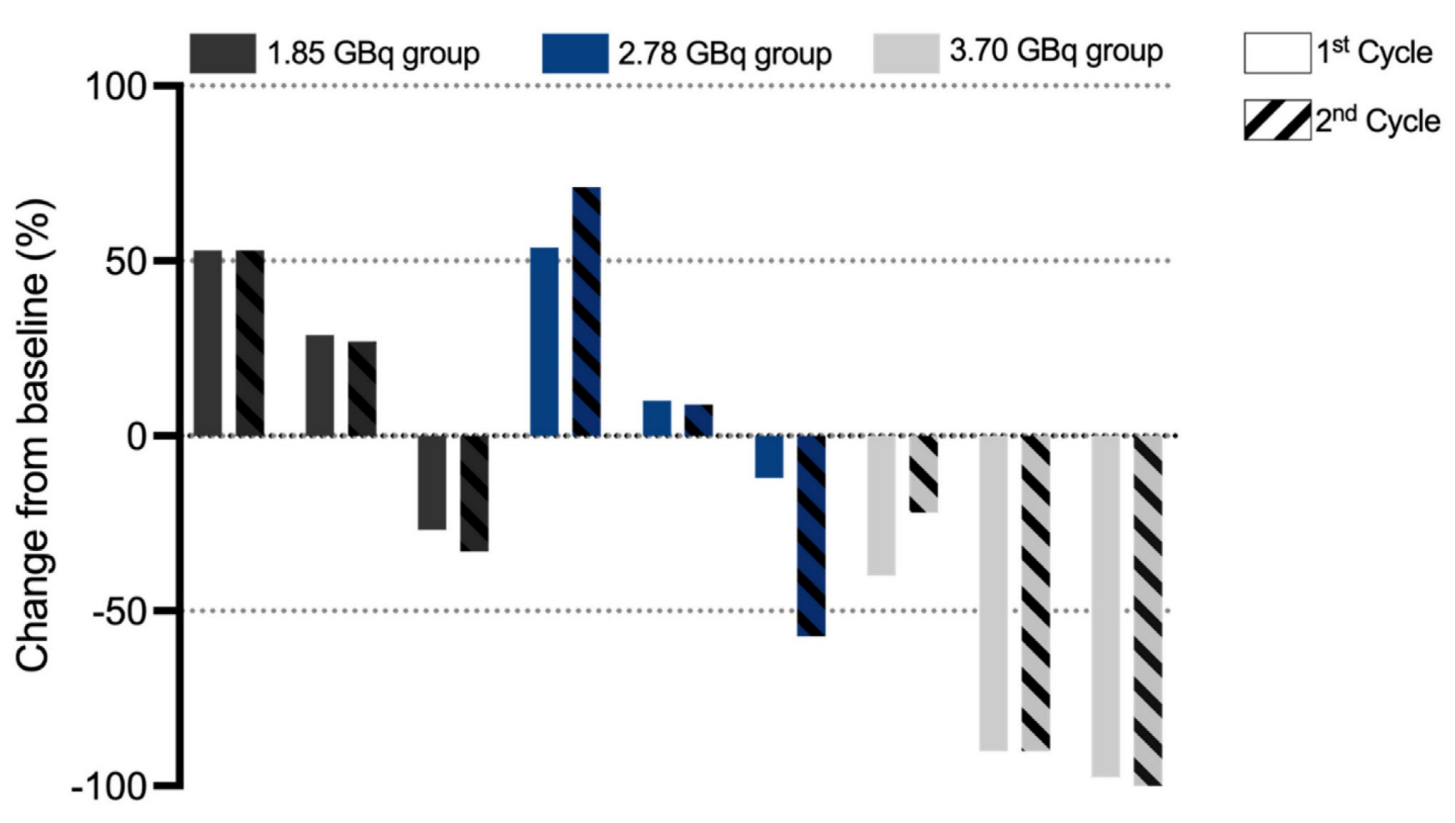
Waterfall plot of the best PSA response in different dose groups after two treatment cycles.

**Figure 4:**
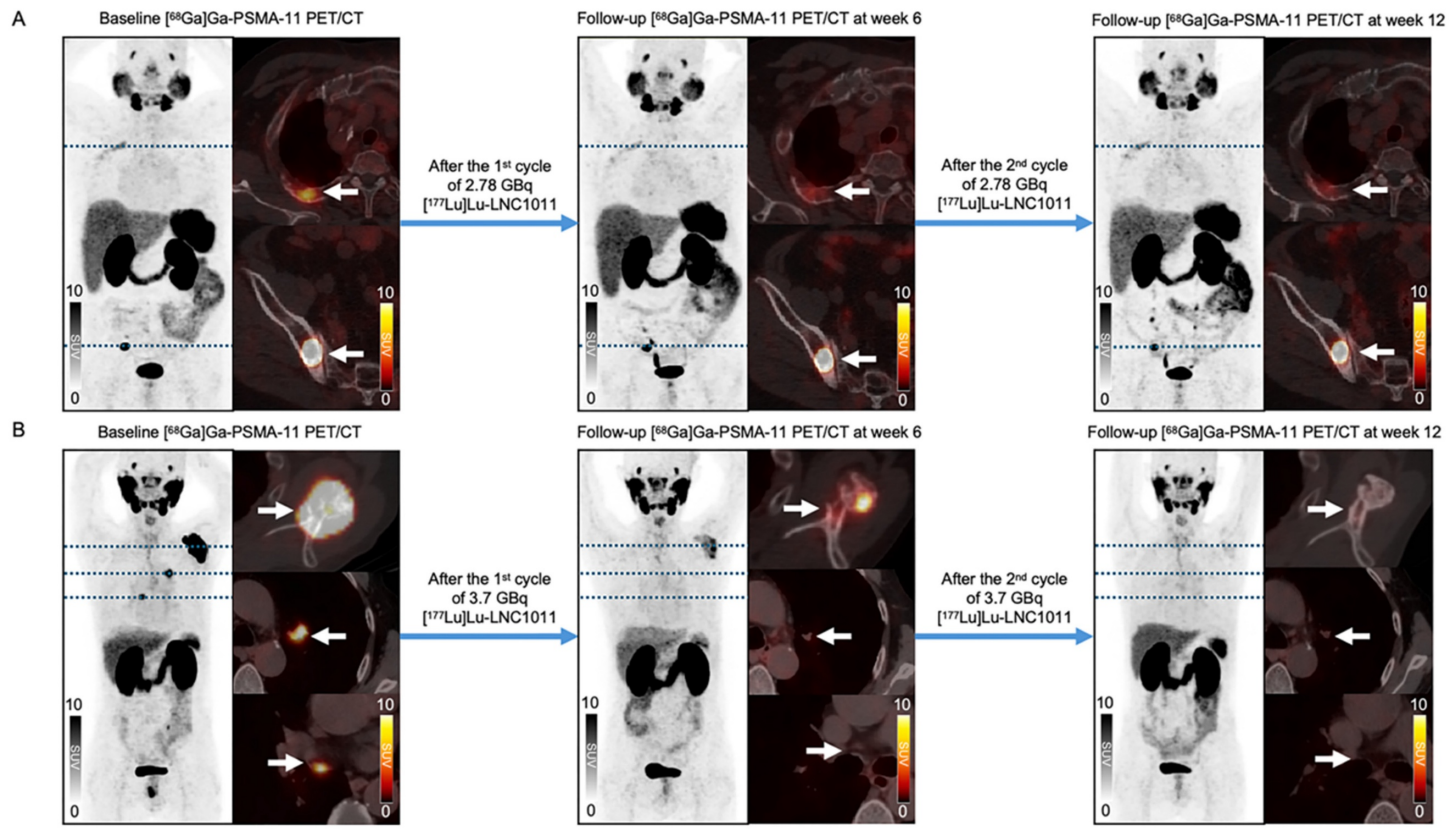
Representative patients' treatment responses: (A) In the 2.78 GBq cohort, a 76-year-old male patient (No. 6) exhibited a bone metastasis lesion in the right iliac. After 2 treatment cycles, the lesion's SUVmax decreased from 62.18 to 37.19 and further to 34.03, accompanied by a decline in PSA levels (from 6.66 to 5.86 to 2.85 ng/mL) and PSMA-VOL reduction (from 7.5 to 6.4 to 5.04 cm³). (B) In the 3.70 GBq dosing cohort, a significant reduction in uptake was observed in the left scapula of a 65-year-old male patient (No. 7), with the SUVmax decreasing from 79.26 to 10.59 to 2.25. The patient's PSA levels changed from 21.1 to 0.52 to 0.013 ng/mL, and PSMA-VOL decreased from 16.18 to 10.98 to 2.31 cm³.

According to PCWG3 criteria, after two treatment cycles, three patients (33.33%, 3/9) exhibited PD, with 2 cases in Activity Group 1 and 1 case in Activity Group 2. SD was observed in 3 patients (33.33%, 3/9), evenly distributed across the three activity groups. 3 patients achieved PR, including 1 case in Activity Group 2 and 2 cases in Activity Group 3. These results demonstrated activity level-dependent therapeutic efficacy, with higher response rates observed in cohorts with escalated activity. The best PSA response after two treatment cycles is shown in **Figure 3**. Following two cycles of [¹⁷⁷Lu]Lu-LNC1011 therapy, as evaluated by RECIP 1.0 for PSMA PET/CT-based assessment and RECIST 1.1 for measurable disease on CT. Therapeutic responses were evaluated using the RECIP 1.0 criteria across the three activity cohorts (1.85 GBq, 2.78 GBq, and 3.70 GBq), which were stratified by treatment cycles. In the 1.85 GBq group, 2 out of 3 patients (66.7%) maintained SD throughout both treatment cycles, while one patient (33.3%) showed an improvement from SD in Cycle 1 to PR in Cycle 2. A similar pattern was observed in the 2.78 GBq cohort, where two patients (66.7%) sustained SD across cycles, and one patient (33.3%) converted from SD to PR in Cycle 2. Notably, the cohort treated with the highest activity (3.70 GBq) demonstrated enhanced efficacy: one patient (33.3%) achieved PR in both cycles, one remained in SD throughout both cycles, and another improved from SD in Cycle 1 to PR in Cycle 2. Overall, this disease control was observed throughout two treatment cycles in three activity groups. In the highest dosage group, the DCR was 100% and the ORR was 66.67%. Of the 9 enrolled patients, 5 had measurable disease at baseline per RECIST 1.1. Among these 5 patients, 4 (80.0%) achieved SD, and 1 (20.0%) attained PR. Representative patient images are shown in **Figure 4**.

## Discussion

In this Phase I study, we evaluated the safety, dosimetry, and initial therapeutic efficacy of the novel PSMA-targeted radioligand [¹⁷⁷Lu]Lu-LNC1011 in patients with mCRPC. Across the escalating dose levels, the results demonstrated that [¹⁷⁷Lu]Lu-LNC1011 exhibited a manageable safety profile and delivered high tumor absorbed radiation doses. Encouraging initial signs of anti-tumor activity were also observed.

Serial multi-time-point whole-body SPECT imaging demonstrated that tumor uptake of [^177^Lu]Lu-LNC1011 was observed as early as 2 h and persisted for up to 168 h (**Figure 1**), highlighting its excellent tumor retention. This kinetic pattern is attributed to the modification of the albumin-binding moiety, aiming to achieve deeper tumor residence than that of conventional PSMA ligands. This tumor uptake and kinetic pattern strongly support the potential of LNC1011 as a therapeutic when labeled with long-lived therapeutic radioisotopes. Dosimetry analysis showed that the absorbed dose of [^177^Lu]Lu-LNC1011 to the salivary glands was lower than the previously reported value of [^177^Lu]Lu-PSMA-617 and [^177^Lu]Lu-PSMA-I&T, while the tumor uptake dose was 3-4 times higher 26. Despite the relatively high renal uptake observed in our trial, no renal functional abnormalities were detected in the three groups. Prior studies have discussed that nephrotoxicity does not always directly correlate with absorbed dose in PSMA RPT 27-29, and the present findings are consistent with this experience. Collectively, the imaging and dosimetry results suggest that [¹⁷⁷Lu]Lu-LNC1011 offers improved tumor retention compared with existing PSMA ligands, while maintaining a favorable safety profile, thereby supporting its further clinical development.

Previous albumin-binding PSMA therapeutic agents have demonstrated significant therapeutic advantages at the expense of increased marrow or salivary gland exposure 30. To ensure treatment safety while preserving tumor retention, [¹⁷⁷Lu]Lu-LNC1011 identified dansylated amino acid (Dan-Phe) as an alternative albumin binder to achieve a balance. Preclinical evidence demonstrates that [¹⁷⁷Lu]Lu-LNC1011 exhibited an approximately 7-8 fold higher tumor uptake compared to [¹⁷⁷Lu]Lu-PSMA-617 22. In the current clinical data, although the cumulative absorbed doses to the kidneys were higher than those of [¹⁷⁷Lu]Lu-PSMA-617, they remained below the threshold of 23 Gy. From the safety perspective, no DLTs were observed during the two treatment cycles. However, in the 3.70 GBq cohort, one patient experienced grade 3 thrombocytopenia during follow-up, which was determined to be treatment-related. Nevertheless, the patient exhibited no signs of bleeding or sequelae during follow-up, and the event did not meet the criteria for a DLT. Guidelines and previous studies indicate that the endpoints for dose-escalation studies in radionuclide therapy differ from those for conventional non-radioactive drugs and can be adapted to specific circumstances, incorporating dosimetric and biological considerations 31, 32. In this study, no DLTs were observed across the dose-escalation groups, supporting the continuation of evaluation, and the single grade 3 thrombocytopenia event in the highest-activity cohort resolved without sequelae. These results support the dansyl modification as a rational design evolution. However, continued monitoring for marrow toxicity remains warranted as cumulative exposures increase in later-phase trials with multiple additional cycles.

In the initial efficacy assessment, PSA reduction and sustained disease stabilisation were observed across all three activity groups. Considering safety, radiation dosimetry, and therapeutic efficacy, our dose-escalation design enabled systematic safety monitoring, evaluation of absorbed doses across escalating activities, and evidence-based determination that 3.70 GBq offers a balanced profile and may be the optimal recommended activity for further expansion.

A limitation of this study is the relatively small sample size, particularly in the dose-escalation cohorts. Furthermore, baseline characteristics (including prior therapy exposure, metastatic patterns, and PSA levels) were imbalanced across the three dosimetric cohorts, and the assessment of activity-dependent efficacy may be limited to only two treatment cycles. Nevertheless, as a Phase I dose-escalation trial, the primary objective was to determine the safety and dosimetric profile of the investigational drug. The analysis was restricted to two treatment cycles, precluding assessment of long-term marrow or renal safety and the durability of response. Nonetheless, these parameters were appropriate for the primary objectives of a Phase I study designed to establish safety, tolerability, and dosimetry. Two cycles were sufficient to capture acute toxicities, characterize organ-specific absorbed doses, and provide preliminary evidence of therapeutic activity. This approach aligns with established ethical standards and methodological expectations for early-phase trials of novel radiopharmaceuticals and supports subsequent multi-cycle investigations. Future research on [^177^Lu]Lu-LNC1011 will focus on large-scale, multicenter clinical trials to further define its long-term safety, optimal dosing, and therapeutic effectiveness. These efforts will optimize the clinical application of [^177^Lu]Lu-LNC1011 and expand its role in personalized precision therapy for mCRPC patients.

## Conclusion

[^177^Lu]Lu-LNC1011 demonstrated early tumor uptake with high tumor absorbed dose and long tumor retention. No dose-limiting toxicities were observed during the dose-escalation trial. Compared with the low-activity group, the 3.70 GBq [^177^Lu]Lu-LNC1011 group demonstrated superior PSA response and imaging outcomes. Considering its safety, dosimetric characteristics, and therapeutic efficacy, the 3.70 GBq activity is deemed suitable for further exploration in large-cohort, multi-cycle treatment studies.

## Supplementary Material

Supplementary methods.

## Figures and Tables

**Table 1:** Characteristics of patients.

Characteristics	1.85 GBq Group	2.78 GBq Group	3.70 GBq Group
Patient no.	n = 3	n = 3	n = 3
Age	72 (69-74)	74 (64-81)	72 (65-78)
ECOG			
0	n = 2	n = 0	n = 0
1	n = 1	n = 3	n = 3
Gleason score	9 (8-9)	9 (9-10)	9 (9-10)
Baseline PSA level (ng/mL)	44.88 (10.23-100)	37.31 (5.27-100)	366.67(21.10-1001.00)
Metastasis			
Lymph node	2	1	2
Bone	3	3	3
Previous therapy			
Prostatectomy	3	1	0
Radiation therapy	1	1	1
Chemotherapy	2	0	1
^223^Ra therapy	0	0	0
LHRH analogs	3	1	3
Bicalutamide	3	2	2
Abiraterone	3	1	1
Enzalutamide	3	0	1

**ECOG**: Eastern Cooperative Oncology Group; **LHRH**: Luteinizing Hormone Releasing Hormone

**Table 2:** Hematotoxicity, hepatotoxicity, and nephrotoxicity before and 6 weeks after [^177^Lu]Lu-LNC1011 RPT, according to CTCAE 5.0.

AEs	Group	Staging	Grade 1~2	Grade 3~4
Anemia	1.85 GBq	Baseline	0	0
		After the first RPT	0	0
		After the second RPT	1 (33.33%)	0
	2.78 GBq	Baseline	3 (100.00%)	0
		After the first RPT	2 (66.67%)	0
		After the second RPT	2 (66.67%)	0
	3.70 GBq	Baseline	2 (66.67%)	0
		After the first RPT	2 (66.67%)	0
		After the second RPT	2 (66.67%)	0
Thrombocytopenia	1.85 GBq	Baseline	0	0
		After the first RPT	0	0
		After the second RPT	0	0
	2.78 GBq	Baseline	0	0
		After the first RPT	0	0
		After the second RPT	0	0
	3.70 GBq	Baseline	0	0
		After the first RPT	1 (33.33%)	0
		After the second RPT	0	1 (33.33%)
Leukopenia	1.85 GBq	Baseline	0	0
		After the first RPT	2 (66.67%)	0
		After the second RPT	2 (66.67%)	0
	2.78 GBq	Baseline	0	0
		After the first RPT	1 (33.33%)	0
		After the second RPT	1 (33.33%)	0
	3.70 GBq	Baseline	0	0
		After the first RPT	0	0
		After the second RPT	0	0
Renal and hepatic insufficiency	1.85 GBq	Baseline	0	0
		After the first RPT	0	0
		After the second RPT	0	0
	2.78 GBq	Baseline	1 (33.33%)	0
		After the first RPT	1 (33.33%)	0
		After the second RPT	1 (33.33%)	0
	3.70 GBq	Baseline	0	0
		After the first RPT	0	0
		After the second RPT	0	0

RPT: Radiopharmaceutical Therapy Cycle interval: Fixed 6-week (42-day) interval between cycles DLT assessment: Last safety labs performed at Day 42 ± 3 of each cycle CTCAE 5.0: Common Terminology Criteria for Adverse Events Version 5.0

**Table 3:** Absorbed dose after intravenous administration of [^177^Lu]Lu-LNC1011.

Target Organ	Dose level 1 (Gy/GBq)		Dose level 2 (Gy/GBq)		Dose level 3 (Gy/GBq)		Whole cohort (Gy/GBq)	
	Average	SD	Average	SD	Average	SD	Average	SD
Adrenals	1.26E-01	1.08E-02	1.21E-01	1.89E-02	1.10E-01	9.40E-03	1.19E-01	1.39E-02
Brain	2.95E-02	2.80E-02	2.47E-02	4.19E-03	1.90E-02	5.72E-03	2.44E-02	1.51E-02
Esophagus	7.42E-02	1.76E-02	7.70E-02	3.27E-02	7.66E-02	1.30E-02	7.59E-02	1.97E-02
Eyes	6.87E-02	1.68E-02	7.03E-02	2.98E-02	7.10E-02	1.22E-02	7.00E-02	1.82E-02
Gallbladder Wall	7.96E-02	1.77E-02	8.13E-02	3.01E-02	8.00E-02	1.26E-02	8.03E-02	1.86E-02
Left colon	7.84E-02	1.67E-02	7.98E-02	2.97E-02	7.88E-02	1.26E-02	7.90E-02	1.82E-02
Small Intestine	7.59E-02	1.72E-02	7.78E-02	3.08E-02	7.72E-02	1.28E-02	7.70E-02	1.88E-02
Stomach Wall	7.56E-02	1.74E-02	7.84E-02	3.22E-02	7.75E-02	1.30E-02	7.72E-02	1.94E-02
Right colon	7.66E-02	1.71E-02	7.82E-02	3.03E-02	7.75E-02	1.27E-02	7.74E-02	1.85E-02
Rectum	7.33E-02	1.73E-02	7.54E-02	3.13E-02	7.56E-02	1.29E-02	7.48E-02	1.90E-02
Heart Wall	2.60E-01	5.69E-02	3.22E-01	2.89E-01	3.05E-01	8.38E-02	2.96E-01	1.56E-01
Kidneys	4.01E+00	6.60E-01	3.41E+00	1.61E+00	2.57E+00	3.25E-01	3.33E+00	1.09E+00
Liver	1.70E-01	4.98E-02	1.65E-01	1.75E-02	1.48E-01	9.29E-03	1.61E-01	2.85E-02
Lungs	8.27E-02	2.67E-02	1.59E-01	6.82E-02	1.15E-01	1.75E-02	1.19E-01	5.01E-02
Pancreas	7.82E-02	1.74E-02	5.27E-02	1.05E-02	6.19E-02	2.23E-02	6.43E-02	1.88E-02
Prostate	/	/	7.60E-02	3.15E-02	7.58E-02	1.28E-02	7.59E-02	2.15E-02
Salivary Glands	2.07E+00	7.93E-01	8.51E-01	2.44E-01	2.19E+00	1.41E+00	1.70E+00	1.04E+00
Red Marrow	9.99E-02	2.35E-02	1.18E-01	4.33E-02	1.21E-01	2.14E-02	1.13E-01	2.86E-02
Osteogenic Cells	1.04E-01	2.42E-02	1.14E-01	4.22E-02	1.16E-01	1.96E-02	1.11E-01	2.68E-02
Spleen	1.27E-01	3.67E-02	3.25E-01	1.55E-01	2.17E-01	1.45E-01	2.23E-01	1.38E-01
Testes	6.93E-02	1.69E-02	7.14E-02	3.06E-02	7.17E-02	1.25E-02	7.08E-02	1.86E-02
Thymus	7.28E-02	1.77E-02	7.64E-02	3.43E-02	7.59E-02	1.33E-02	7.50E-02	2.05E-02
Thyroid	7.10E-02	1.74E-02	7.34E-02	3.15E-02	7.36E-02	1.26E-02	7.27E-02	1.91E-02
Urinary Bladder Wall	2.38E-01	6.71E-02	2.83E-01	1.74E-01	2.60E-01	2.08E-02	2.61E-01	9.57E-02
Total Body	1.00E-01	1.79E-02	1.02E-01	3.15E-02	9.81E-02	1.25E-02	1.00E-01	1.92E-02
Effective Dose (mSv/MBq)	1.32E-01	1.76E-02	1.30E-01	2.94E-02	1.28E-01	1.66E-02	1.30E-01	1.91E-02

## Data Availability

The datasets generated during and/or analyzed during the current study are available from the corresponding author on reasonable request.

## References

[1] Siegel RL, Giaquinto AN, Jemal A (2024). Cancer statistics, 2024. CA Cancer J Clin.

[2] Sung H, Ferlay J, Siegel RL, Laversanne M, Soerjomataram I, Jemal A (2021). Global Cancer Statistics 2020: GLOBOCAN Estimates of Incidence and Mortality Worldwide for 36 Cancers in 185 Countries. CA Cancer J Clin.

[3] Evans JC, Malhotra M, Cryan JF, O'Driscoll CM (2016). The therapeutic and diagnostic potential of the prostate specific membrane antigen/glutamate carboxypeptidase II (PSMA/GCPII) in cancer and neurological disease. Br J Pharmacol.

[4] Oprea-Lager DE, MacLennan S, Bjartell A, Briganti A, Burger IA, de Jong I (2024). European Association of Nuclear Medicine Focus 5: Consensus on Molecular Imaging and Theranostics in Prostate Cancer. Eur Urol.

[5] Hofman MS, Violet J, Hicks RJ, Ferdinandus J, Thang SP, Akhurst T (2018). [(177)Lu]-PSMA-617 radionuclide treatment in patients with metastatic castration-resistant prostate cancer (LuPSMA trial): a single-centre, single-arm, phase 2 study. Lancet Oncol.

[6] Hofman MS, Emmett L, Sandhu S, Iravani A, Joshua AM, Goh JC (2021). [(177)Lu]Lu-PSMA-617 versus cabazitaxel in patients with metastatic castration-resistant prostate cancer (TheraP): a randomised, open-label, phase 2 trial. Lancet.

[7] Sartor O, de Bono J, Chi KN, Fizazi K, Herrmann K, Rahbar K (2021). Lutetium-177-PSMA-617 for Metastatic Castration-Resistant Prostate Cancer. N Engl J Med.

[8] Hennrich U, Eder M (2022). [(177)Lu]Lu-PSMA-617 (Pluvicto(TM)): The First FDA-Approved Radiotherapeutical for Treatment of Prostate Cancer. Pharmaceuticals (Basel).

[9] Kratochwil C, Fendler WP, Eiber M, Hofman MS, Emmett L, Calais J (2023). Joint EANM/SNMMI procedure guideline for the use of (177)Lu-labeled PSMA-targeted radioligand-therapy ((177)Lu-PSMA-RLT). Eur J Nucl Med Mol Imaging.

[10] Liu Z, Chen X (2016). Simple bioconjugate chemistry serves great clinical advances: albumin as a versatile platform for diagnosis and precision therapy. Chem Soc Rev.

[11] Muller C, Struthers H, Winiger C, Zhernosekov K, Schibli R (2013). DOTA conjugate with an albumin-binding entity enables the first folic acid-targeted 177Lu-radionuclide tumor therapy in mice. J Nucl Med.

[12] Kuo HT, Zhang Z, Zhang C, Merkens H, Tan R, Wong A (2023). Lys-urea-Aad, Lys-urea-Cmc and Lys-urea-Cms as potential pharmacophores for the design of PSMA-targeted radioligands to reduce off-target uptake in kidneys and salivary glands. Theranostics.

[13] Boinapally S, Alati S, Jiang Z, Yan Y, Lisok A, Singh R (2023). Preclinical Evaluation of a New Series of Albumin-Binding (177)Lu-Labeled PSMA-Based Low-Molecular-Weight Radiotherapeutics. Molecules.

[14] Deberle LM, Benesova M, Umbricht CA, Borgna F, Buchler M, Zhernosekov K (2020). Development of a new class of PSMA radioligands comprising ibuprofen as an albumin-binding entity. Theranostics.

[15] Benesova M, Umbricht CA, Schibli R, Muller C (2018). Albumin-Binding PSMA Ligands: Optimization of the Tissue Distribution Profile. Mol Pharm.

[16] Wang Z, Tian R, Niu G, Ma Y, Lang L, Szajek LP (2018). Single Low-Dose Injection of Evans Blue Modified PSMA-617 Radioligand Therapy Eliminates Prostate-Specific Membrane Antigen Positive Tumors. Bioconjug Chem.

[17] Zang J, Fan X, Wang H, Liu Q, Wang J, Li H (2019). First-in-human study of (177)Lu-EB-PSMA-617 in patients with metastatic castration-resistant prostate cancer. Eur J Nucl Med Mol Imaging.

[18] Zang J, Liu Q, Sui H, Wang R, Jacobson O, Fan X (2020). (177)Lu-EB-PSMA Radioligand Therapy with Escalating Doses in Patients with Metastatic Castration-Resistant Prostate Cancer. J Nucl Med.

[19] Wang G, Zang J, Jiang Y, Liu Q, Sui H, Wang R (2023). A Single-Arm, Low-Dose, Prospective Study of (177)Lu-EB-PSMA Radioligand Therapy in Patients with Metastatic Castration-Resistant Prostate Cancer. J Nucl Med.

[20] Zang J, Wang G, Zhao T, Liu H, Lin X, Yang Y (2024). A phase 1 trial to determine the maximum tolerated dose and patient-specific dosimetry of [(177)Lu]Lu-LNC1003 in patients with metastatic castration-resistant prostate cancer. Eur J Nucl Med Mol Imaging.

[21] Wen X, Xu P, Zeng X, Liu J, Du C, Zeng X (2023). Development of [(177)Lu]Lu-LNC1003 for radioligand therapy of prostate cancer with a moderate level of PSMA expression. Eur J Nucl Med Mol Imaging.

[22] Yang H, Wang J, Wen X, Guo H, Jakobsson V, Zhao T Dansylated Amino Acid-Modified Long-Acting PSMA Derivatives (68)Ga/(177)Lu-LNC1011 as Prostate Cancer Theranostics. *J Nucl Med*. 2025: 10.2967/jnumed.124.268959.

[23] Scher HI, Morris MJ, Stadler WM, Higano C, Basch E, Fizazi K (2016). Trial Design and Objectives for Castration-Resistant Prostate Cancer: Updated Recommendations From the Prostate Cancer Clinical Trials Working Group 3. J Clin Oncol.

[24] Eisenhauer EA, Therasse P, Bogaerts J, Schwartz LH, Sargent D, Ford R (2009). New response evaluation criteria in solid tumours: revised RECIST guideline (version 1.1). Eur J Cancer.

[25] Gafita A, Djaileb L, Rauscher I, Fendler WP, Hadaschik B, Rowe SP (2023). Response Evaluation Criteria in PSMA PET/CT (RECIP 1.0) in Metastatic Castration-resistant Prostate Cancer. Radiology.

[26] Ells Z, Grogan TR, Czernin J, Dahlbom M, Calais J (2024). Dosimetry of [(177)Lu]Lu-PSMA-Targeted Radiopharmaceutical Therapies in Patients with Prostate Cancer: A Comparative Systematic Review and Metaanalysis. J Nucl Med.

[27] Strosberg J, Hofman MS, Al-Toubah T, Hope TA (2024). Rethinking Dosimetry: The Perils of Extrapolated External-Beam Radiotherapy Constraints to Radionuclide Therapy. J Nucl Med.

[28] Strosberg JR, Caplin ME, Kunz PL, Ruszniewski PB, Bodei L, Hendifar A (2021). (177)Lu-Dotatate plus long-acting octreotide versus high-dose long-acting octreotide in patients with midgut neuroendocrine tumours (NETTER-1): final overall survival and long-term safety results from an open-label, randomised, controlled, phase 3 trial. Lancet Oncol.

[29] Steinhelfer L, Lunger L, Cala L, Pfob CH, Lapa C, Hartrampf PE (2024). Long-Term Nephrotoxicity of (177)Lu-PSMA Radioligand Therapy. J Nucl Med.

[30] Baum RP, Kulkarni HR (2012). THERANOSTICS: From Molecular Imaging Using Ga-68 Labeled Tracers and PET/CT to Personalized Radionuclide Therapy - The Bad Berka Experience. Theranostics.

[31] van Gerven J, Bonelli M (2018). Commentary on the EMA Guideline on strategies to identify and mitigate risks for first-in-human and early clinical trials with investigational medicinal products. Br J Clin Pharmacol.

[32] Kratochwil C, Bruchertseifer F, Rathke H, Hohenfellner M, Giesel FL, Haberkorn U (2018). Targeted alpha-Therapy of Metastatic Castration-Resistant Prostate Cancer with (225)Ac-PSMA-617: Swimmer-Plot Analysis Suggests Efficacy Regarding Duration of Tumor Control. J Nucl Med.

