# Supplementary material for: Phase I dose-escalation study of [¹⁷⁷Lu]Lu-LNC1011, a long-circulating dansyl-modified PSMA theranostics, in metastatic castration-resistant prostate cancer
Source: Theranostics. 2026 Mar 4;16(10):5175–84. doi: 10.7150/thno.128143 (PMC13080328; doi:10.7150/thno.128143)
Supplement: Supplementary file 1 — Supplementary methods. [file thnov16p5175s1.pdf]

Supplemental Materials

Phase I Dose-Escalation Study of [^{177}Lu]Lu-D-Dan-Phe-PSMA ([^{177}Lu]Lu-LNC1011): A Long-Circulating Dansyl-Modified PSMA Theranostic in Metastatic Castration-Resistant Prostate Cancer

Jiarou Wang[1*], Rongxi Wang[1*], Jialin Xiang[1*], Hongzhang Yang[2], Tianzhi Zhao[2,3,5,6], Feng Guo[4], Yingkui Liang[4], Zhaohui Zhu[1#], Jingjing Zhang[2,3,5,6#]

Eligibility

This study enrolled mCRPC patients who were PSMA-positive and met the following criteria: 1) A rise of at least 25% in serum Prostate-Specific Antigen (PSA) levels on two consecutive measurements (separated by at least two weeks) from the nadir or the appearance of new lesions on imaging, despite hormonal therapy resulting in serum testosterone levels of < 50 ng/dl (< 1.7 nmol/l); 2) Prior treatment with at least one novel androgen-axis drug (e.g., enzalutamide or abiraterone); 3) Hematologic, hepatic, and renal function indicators meet the requirements (white blood cell count > 2.5 × 10^9/L, platelet count > 75 × 10^9/L, hemoglobin > 9.0 g/dL, serum albumin > 25 g/L, total bilirubin (< 60 µmol/L, glomerular filtration rate > 40 mL/min, and serum creatinine (< 150 µmol/L), with an Eastern Cooperative Oncology Group (ECOG) performance status score of ≤ 2; 4) Patients underwent dual scans with [^{68}Ga]Ga-PSMA-11 and [^{18}F]FDG PET/CT. PET eligibility criteria for the trial included PSMA-positive disease with a maximum standardized uptake value (SUV_{max}) of at least 20 at a disease site and greater than 10 at all other measurable metastatic disease sites, with no sites showing discordant [^{18}F]FDG-positive and PSMA-negative findings.

Radiopharmaceuticals

For the radiolabeling process of [^{177}Lu]Lu-LNC1011, 50 µg of LNC1011 was combined with [^{177}Lu]$LuCl_3$ (0.74–1.11 GBq in 0.04 M HCl). The pH was then adjusted to 5.5 using 0.5

M ammonium acetate, followed by heating the mixture at 95 °C for 30 minutes. The radiochemical yield, purity, and molar activity were determined using a Dionex Ulti-Mate 3000 high-performance liquid chromatography system (Thermo Scientific). This system was equipped with a radio-scanner (MSFC1-00220, Eckert & Ziegler) and an analytical C-18 reversed-phase column (4.6 × 250 mm, 5 μm, 120 Å, Thermo). The HPLC conditions employed a mobile phase consisting of 0.1% trifluoroacetic acid (TFA) in water (A) and 0.1% TFA in acetonitrile (B), with a gradient from 5% B to 95% B over 20 minutes at a flow rate of 1 mL/min.

Imaging and dosimetry

The intravenous [^{68}Ga]Ga-PSMA-11 dose, ranging from 1.85 to 2.22 MBq/Kg, was administered prior to PET/CT imaging on a Polestar m660 scanner 50-60 minutes post-injection. Preceding the PET/CT, a low-dose CT scan for attenuation and anatomical referencing was performed from the skull vertex to the mid-thigh at 120 kV and 30-50 mAs. The whole-body scan was acquired in 5-6 bed positions, each for 2 minutes, with images reconstructed using the ordered-subset expectation maximization algorithm. Pharmacokinetic (PK) analysis focused on quantifying the radiopharmaceutical's biodistribution kinetics, including Time-activity curves (TACs) derived from serial imaging, effective half-lives in organs/tumors and residence times calculated from TAC integration.

During the dose-escalation phase, we conducted multiple planar whole-body scans and collected blood samples at various time points for pharmacokinetic analysis in the first cycle of 9 patients. Whole-body (WB) scans were performed at 2, 4, 24, 48, 72, 120, and 168 hours following intravenous administration of [^{177}Lu]Lu-LNC1011 using a Philips Precedence scanner (Philips Healthcare, Andover, Massachusetts, USA). The scans utilized a medium-energy general-purpose collimator, a 20% energy window, a peak at 208 keV, a scan speed of 15 cm/min for whole-body imaging, and 32 frames with a 40-second exposure time per frame for each tomographic scan. Extra SPECT/CT imaging was conducted at 24 hours post-injection

for each patient using the same scanner, incorporating a low-dose CT (120 kV, 35 mA, 512 × 512 matrix, 3-mm layer, 70 cm field of view) from the second rib to the proximal thigh for attenuation correction and anatomical localization. Blood samples were collected prior to each SPECT scan. Additionally, an additional blood sample was collected 5 minutes post-injection and the activity was calculated using a gamma counter.

Dosimetry calculations were performed using the Hybrid-Dosimetry software (Hermes Medical Solutions, Sweden) and OLINDA/EXM (version 2.2.0). Absorbed doses were measured in the brain, salivary glands, thyroid, cardiac content, lungs, liver, kidneys, spleen, pancreas, L2-L4 lumbar vertebrae, gastric content, and bladder content. Red marrow doses were calculated using a 3D volume analysis of the L2-L4 vertebrae, representing approximately 6.7% of the total bone marrow. Target organs and regions of interest (ROI) were delineated by two experienced nuclear medicine physicians, selecting appropriate lesions for dosimetry measurements. Patients were advised not to empty their bladders prior to the initial whole-body scan, establishing the whole-body counts from this scan as the reference for 100% of the administered activity.

Treatment regimen and follow-up

Patients received intravenous hydration (3,000 mL of 0.9% NaCl) starting 10 min before administration. [^{177}Lu]Lu-LNC1011 was diluted in 30 mL of normal saline was co-administered slowly in an intravenous infusion for 10 min. All patients underwent a complete blood count, liver and kidney, and PSA measurements four weeks after drug administration. Patient survival (survival status, weight, disease-related symptoms) and general condition (AEs such as fatigue, nausea, vomiting, dry mouth, etc.) were followed up by telephone every two weeks.

The effective half-lives in other organs

The effective half-life of [^{177}Lu]Lu-LNC1011 in different organs was as follows: 98.50 ± 22.97 hours in the kidney, 38.56 ± 9.87 hours in the liver, 75.54 ± 18.46 hours in the parotid glands, 34.00 ± 8.77 hours in the red bone marrow, and 68.56 ± 42.70 hours in the spleen.
